# Resistant hypertension: consensus document from the Korean society of hypertension

**DOI:** 10.1186/s40885-023-00255-4

**Published:** 2023-11-01

**Authors:** Sungha Park, Jinho Shin, Sang Hyun Ihm, Kwang-il Kim, Hack-Lyoung Kim, Hyeon Chang Kim, Eun Mi Lee, Jang Hoon Lee, Shin Young Ahn, Eun Joo Cho, Ju Han Kim, Hee-Taik Kang, Hae-Young Lee, Sunki Lee, Woohyeun Kim, Jong-Moo Park

**Affiliations:** 1 Division of Cardiology, Severance Cardiovascular Hospital, Integrative Research Center for Cerebrovascular and Cardiovascular Diseases, Yonsei University College of Medicine, Seoul, Republic of Korea; 2 Division of Cardiology, Department of Internal Medicine, Hanyang University Seoul Hospital, Seoul, South Korea; 3 Division of Cardiology, Department of Internal Medicine, Bucheon St. Mary’s Hospital, College of Medicine, The Catholic University of Korea, Bucheon, Republic of Korea; 4 Catholic Research Institute for Intractable Cardiovascular Disease, College of Medicine, The Catholic University of Korea, Bucheon St. Mary’s Hospital327 Sosa-Ro, Wonmi-guGyunggi-do, Bucheon-si, 14647 Republic of Korea; 5 Department of Internal Medicine, Seoul National University Bundang Hospital, Seoul National University College of Medicine, Seongnam, Republic of Korea; 6 Division of Cardiology, Department of Internal Medicine, Boramae Medical Center, Seoul National University College of Medicine, Seoul, Korea; 7 Department of Preventive Medicine, Yonsei University College of Medicine, Seoul, Republic of Korea; 8 Division of Cardiology, Department of Internal Medicine, Wonkwang University Sanbon Hospital, Wonkwang University College of Medicine, Gunpo, Republic of Korea; 9 Department of Internal Medicine, Kyungpook National University Hospital, Daegu, South Korea; 10 School of Medicine, Kyungpook National University, Daegu, South Korea; 11 Division of Nephrology, Department of Internal Medicine, Korea University Guro Hospital, Seoul, South Korea; 12 Division of Cardiology, Department of Internal Medicine, The Catholic University of Korea, Seoul, Korea; 13 Department of Cardiovascular Medicine, Chonnam National University Hospital, Gwangju, Korea; 14 Department of Family Medicine, Severance Hospital, Yonsei University College of Medicine, Seoul, Republic of Korea; 15 Department of Internal Medicine, Seoul National University Hospital, Seoul National University College of Medicine, Seoul, Republic of Korea; 16 Hallym University, Dongtan Hospital, Gyeonggi-do, Korea; 17 Division of Cardiology, Department of Internal Medicine, Hanyang University Seoul Hospital, Seoul, Korea; 18 Department of Neurology, Uijeongbu Eulji Medical Center, Eulji University, Uijeongbu, South Korea

**Keywords:** Ambulatory blood pressure monitoring, Home blood pressure monitoring, Hypertension, Refractory hypertension, Resistant hypertension

## Abstract

Although reports vary, the prevalence of true resistant hypertension and apparent treatment-resistant hypertension (aTRH) has been reported to be 10.3% and 14.7%, respectively. As there is a rapid increase in the prevalence of obesity, chronic kidney disease, and diabetes mellitus, factors that are associated with resistant hypertension, the prevalence of resistant hypertension is expected to rise as well. Frequently, patients with aTRH have pseudoresistant hypertension [aTRH due to white-coat uncontrolled hypertension (WUCH), drug underdosing, poor adherence, and inaccurate office blood pressure (BP) measurements]. As the prevalence of WUCH is high among patients with aTRH, the use of out-of-office BP measurements, both ambulatory blood pressure monitoring (ABPM) and home blood pressure monitoring (HBPM), is essential to exclude WUCH. Non-adherence is especially problematic, and methods to assess adherence remain limited and often not clinically feasible. Therefore, the use of HBPM and higher utilization of single-pill fixed-dose combination treatments should be emphasized to improve drug adherence. In addition, primary aldosteronism and symptomatic obstructive sleep apnea are quite common in patients with hypertension and more so in patients with resistant hypertension. Screening for these diseases is essential, as the treatment of these secondary causes may help control BP in patients who are otherwise difficult to treat. Finally, a proper drug regimen combined with lifestyle modifications is essential to control BP in these patients.

**Graphical Abstract:**

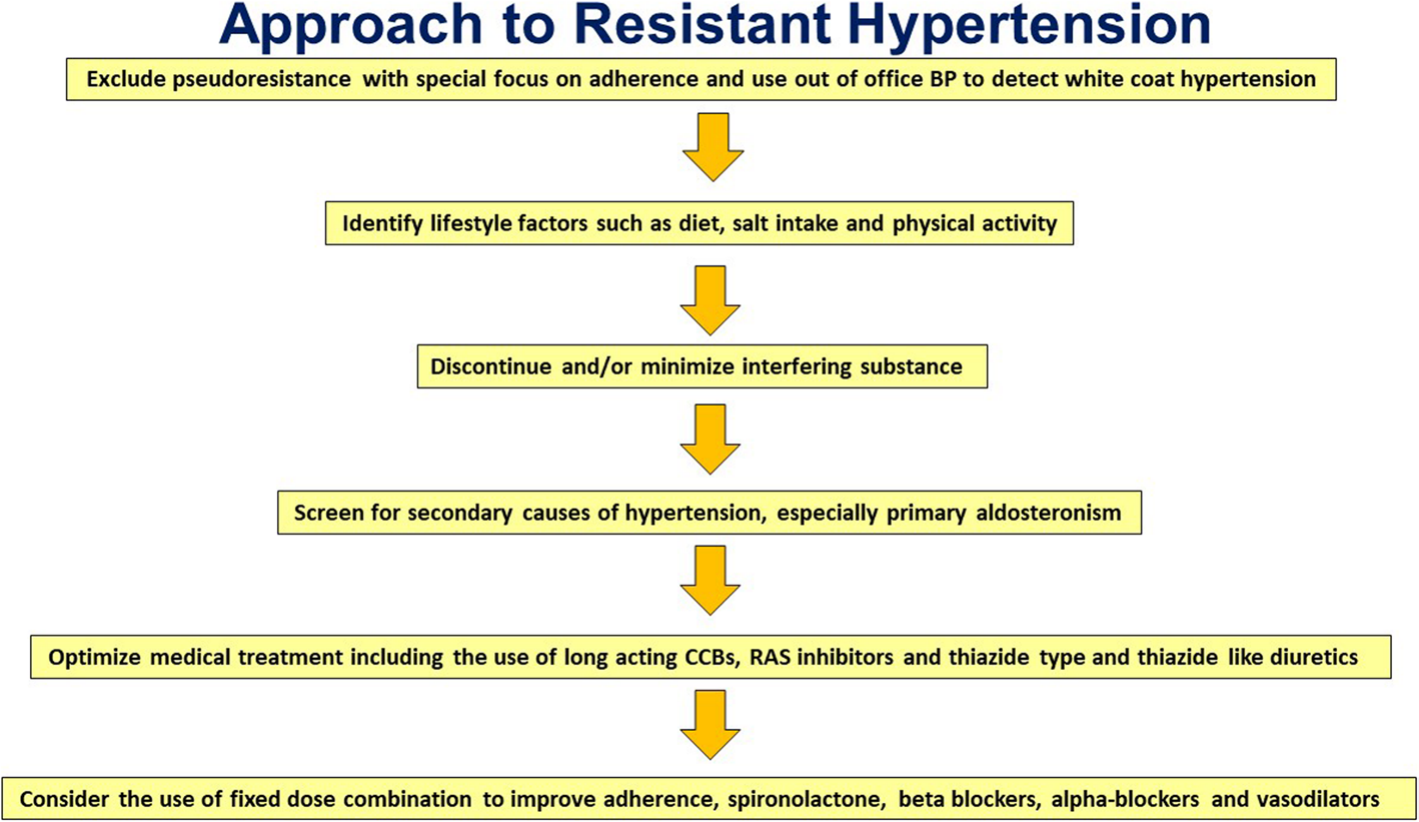

## Introduction

High blood pressure (BP) is associated with increased cardiovascular complications, regardless of the type of prescribed antihypertensive medications [1]. BP reduction at any level has shown a 10% reduction in cardiovascular events per 5 mmHg reduction in systolic BP. In addition to population-based strategies for identifying patients with untreated hypertension and introducing antihypertensive therapy, BP reduction to recommended levels is important for reducing cardiovascular risk and optimizing antihypertension treatment.

In Korea, the overall control rate of hypertension is less than half of the hypertensive population, and the control rate in patients treated for hypertension is approximately 70% [2]. Patients with resistant hypertension (RH) are particularly challenging in terms of the reasons for their reduced response to multiple antihypertensive medications, clinical presentation, specific etiology, prognosis, and management. Understanding the underlying etiology and pathophysiology of uncontrolled hypertension treated with multiple antihypertensive medications will help achieve a breakthrough in the stagnant BP control rate [3]. Accordingly, the Korean Society of Hypertension has published an expert consensus on the definition, epidemiology, etiology, diagnosis, and management of RH.

### Definition of resistant hypertension

RH is defined as the failure to achieve the target BP despite the use of ≥3 antihypertensive drugs, commonly including dihydropyridine calcium channel blockers(CCBs), renin-angiotensin system (RAS) inhibitors, and diuretics, or the need for treatment with ≥4 antihypertensive medications to achieve the target BP [4, 5]. Patients who fail to achieve the target BP despite the use of five or more antihypertensive medications, ideally including thiazide-like diuretics and spironolactone, are defined as having treatment-refractory hypertension [6, 7]. The target BP should be in accordance with the current guidelines. For example, the threshold for diagnosis is 130/80 mmHg according to the American College of Cardiology/American Heart Association guidelines [4]. For Korean patients, the threshold may differ based on underlying risk factors [5, 8]. Previous studies included patients who were diagnosed with hypertension based on office BP as well as patients who would be categorized as having pseudoresistant hypertension. Therefore, the term “apparent treatment RH” (aTRH) should be used for patients in whom pseudoresistant hypertension has not been ruled out. The causes of pseudoresistant hypertension include white-coat uncontrolled hypertension (WUCH), non-adherence, poorly measured office BP, or undertreatment [4]. For an accurate diagnosis of RH, out-of-office BP monitoring, such as 24-hour ambulatory blood pressure monitoring (ABPM) and/or home blood pressure monitoring (HBPM), should be performed to rule out WUCH. This is essential because WUCH impacts as many as 1/3 of patients with aTRH [9]. Moreover, the possibility of non-adherence needs to be ruled out, as it is common, especially with polypharmacy and a higher number of anti- hypertensive medications [10]. Finally, antihypertensive medications, including thiazide-like or thiazide-type diuretics, must be titrated to maximally tolerated doses before making a diagnosis.

### Epidemiology of resistant hypertension

The reported prevalence of RH varies significantly according to specific studies. In the 2018 AHA scientific statement, the prevalence of RH among all patients with hypertension was 12-15% based on population studies and 15-18% for clinic based reports [4]. In a pooled analysis of 3,207,911 patients with hypertension, the prevalence rates of true and aTRH were 10.3% and 14.7%, respectively [11]. Age, higher baseline BP, obesity, excessive salt ingestion, chronic kidney disease (CKD) and diabetes mellitus (DM) were associated with a higher risk of RH [12]. The prevalence of RH increases with age. Several factors contribute to the higher risk of RH in older adults [13, 14]. They include age-related vascular changes (i.e., vascular stiffness), neurohormone imbalances, multiple comorbidities (including kidney disease, obesity, and diabetes), poor adherence to medication or polypharmacy, and insufficient lifestyle modifications (especially high-salt diets) [13, 14].

Noubiap et al. analyzed the prevalence of aTRH by region [11]. They found that the prevalence of aTRH did not differ by region, with the prevalence of RH in the Americas, Asia, and Europe being 14.8%, 14.7%, and 14.8%, respectively. The prevalence of treatment RH in cohorts that underwent out-of-office BP monitoring tended to be lower than that reported previously. In a Korean study that recruited 3088 patients with essential hypertension from 247 primary care physicians, the prevalence of RH was 7.9% [15]. In another study involving 16,284 Korean patients with hypertension who underwent ABPM at a single tertiary referral hospital, the overall prevalence of RH was 10.1%. Within this group, the prevalence of RH (excluding refractory hypertension) was 9.2%, and refractory hypertension accounted for 0.9% of the cases [16]. The prevalence of RH defined by home BP was recently reported in an analysis of the Japan Morning Surge Home BP (J-HOP) study. Using the average of home BP threshold of ≥135/85 mmHg and ≥130/80 mmHg, the prevalence of RH was 10.7% and 12.5%, respectively [17].

RH is associated with an adverse prognosis compared with patients without RH. In a retrospective cohort of 205,750 patients with incident hypertension over a median follow-up of 3.8 years, RH was associated with an approximately 50% higher risk of cardiovascular events[hazard ration (HR):1.47, 95% confidence interval (CI):1.33-1.62] [18]. In a post hoc analysis of 14,684 patients from the Antihypertensive and Lipid-Lowering Treatment to Prevent Heart Attack Trial (ALLHAT), 1870 patients were defined as having aTRH. The results showed that aTRH was associated with an increased risk of coronary heart disease(HR:1.47, 95% CI:1.26-1.71), cardiovascular disease(HR:1.46, 95% CI:1.29-1.64) and end stage renal disease(HR:1.95, 95% CI:1.26-1.71) [19]. In a retrospective analysis of 16,284 Korean patients who underwent ABPM, RH was associated with a 62% higher risk of cardiovascular mortality(HR:1.62, 95% CI:1.16-2.26) [16]. Similarly, RH based on home BP measurement is associated with an adverse prognosis. In an analysis of 4,261 patients with hypertension from the J-HOP study with a median follow-up of 6.2 years, the adjusted HRs of uncontrolled treatment RH were 2.02(1.38-2.94) and 1.81(1.23-2.65) when using a home BP threshold of 135/85 mmHg and 130/80 mmHg, respectively [17]. Use of out-of-office BP monitoring, such as ABPM or HBPM, for the diagnosis and treatment of RH is important, as it can better predict cardiovascular outcomes in patients with RH [20].

### Evaluation of etiology

#### Diagnosis of resistant hypertension

RH can be diagnosed by ruling out pseudoresistance, which is defined as a lack of BP control despite appropriate treatment in patients who do not have RH. Several factors, such as improper BP measurement, the “white-coat effect,” medication non-adherence, and treatment inertia, contribute to pseudoresistance. According to current guidelines, accurate BP measurement is the cornerstone of RH diagnosis [21, 22]. The “white-coat effect” should be excluded when using ABPM or HBPM [23]. Medication non-adherence is common in patients with aTRH [24]. Treatment inertia is one reason why a large subset of patients do not achieve their BP target. Between 2007 and 2010, less than 50% of patients were prescribed an optimal antihypertensive regimen in the United States [23]. Therefore, a careful evaluation to exclude these factors should be performed before diagnosing RH. It is also important to rule out the possibility of pseudoresistance due to the white-coat effect in the diagnosis of RH in older adults, as this phenomenon is more common [25].

### Lifestyle factors contributing resistant hypertension (Table 1)

**Table 1:** Factors causing high blood pressure in resistant hypertension

**Poor medication adherence**
**Lifestyle factors**
Alcohol
Smoking
Obesity
Physical inactivity
High salt intake
Increased stress level
Sleep disturbance
Obstructive sleep apnea
**Medications**
NSAIDs
Glucocorticoids, mineralocorticoids
Oral contraceptives, hormone replacement
Sympathomimetic agents (e.g., decongestants, diet pills)
Cyclosporine, tacrolimus
VEGF inhibitors
Antidepressants
Erythropoietin
Natural licorice
Herbal compounds

*NSAIDs* Nonsteroidal anti-inflammatory drugs, *VEGF* Vascular endothelial growth factor

#### Obesity

Increased adiposity is associated with elevated BP [26]. Obesity-related hypertension is associated with increased salt sensitivity, increased sympathetic nervous system activity, activation of the renin-angiotensin-aldosterone system(RAS), and aldosterone secretion by adipose tissue [26, 27]. In NHANES (National Health and Nutrition Examination Survey), body mass index ≥30 kg/m^2^ doubled the risk to have aTRH [28]. However, no current guidelines recommend the use of specific drugs in patients with obesity.

#### Dietary sodium

Dietary sodium increases BP, particularly in certain groups that are more salt-sensitive or have CKD [29]. Despite large person-to-person variations in salt sensitivity, vascular dysfunction, arterial stiffness, sympathetic activation, impaired renin-angiotensin axis suppression, mineralocorticoid receptor activation, and immune cell modulation are suggested as possible mechanisms [30, 31]. One study showed that reducing dietary sodium from 250 mmol (5.75g)/day to 50 mmol (1.15g)/day for 1-week lowered office systolic BP by 22.7 mmHg (P=0.008) in patients with RH [32]. Patients should be advised to adhere to the DASH (Dietary Approaches to Stop Hypertension) diet with less than 2 g of sodium/day (5 g of table salt) to effectively lower BP [29].

#### Physical inactivity

Physical inactivity is independently associated with BP elevation and risk of hypertension [33]. However, it is uncertain whether sedentary life itself is independently related to RH because of a paucity of data [34]. Recently, a small randomized trial (*n*=50) showed that a thrice-weekly treadmill exercise program for 8–12 weeks lowered daytime systolic ambulatory BP by 5.9 mmHg (*P*=0.03) in patients with RH [34]. Patients with RH should engage in at least 150 min/week of moderate-intensity aerobic exercise or 75 min/week of vigorous-intensity aerobic exercise.

#### Alcohol

Regular alcohol consumption is associated with a BP elevation of 1 mmHg for every 10 g of alcohol consumed (≈1 standard drink) [35]. Heavy alcohol use may be associated with aTRH, since alcohol may interfere with the pharmacologic action of antihypertensive agents [36].

#### Smoking

Nicotine from cigarettes, vaping fluids, and smokeless tobacco increases BP [37]. To reduce cardiovascular events, smoking cessation is recommended for patients with RH.

#### Medication-related resistant hypertension

Some classes of medications can increase BP, including anticonvulsants, antidepressants, antipsychotics, antiemetics, antineoplastic agent, calcineurin inhibitors, contraceptives, erythropoietin, glucocorticoids, mineralocorticoids, nonsteroidal anti-inflammatory drugs (NSAIDs), glucocorticoids, estrogen-containing oral contraceptives and hormone replacement, sympathomimetic amines (pseudoephedrine, ephedrine, cocaine, amphetamine), vascular endothelial growth factor inhibitors, erythropoietin-stimulating agents, immunosuppressive agents (cyclosporine, tacrolimus), anticancer drugs(vascular endothelial growth factor inhibitors, tyrosine kinase inhibitors and proteasome inhibitors), serotonin-norepinephrine reuptake inhibitors, and dietary supplements (ginseng, licorice) [4, 38, 39]. The effect of these drugs on increasing BP varies from person to person, ranging from little to no effect to severely elevated BP. As some medications such as NSAIDs, cold medications, and oral contraceptives can be purchased and taken by patients without a prescription, medication reconciliation is an important process in the management of patients with hypertension [40].

#### Pseudopheochromocytoma

After true pheochromocytoma has been ruled out as the cause of the sudden increase in BP, pseudopheochromocytoma should be considered. Pseudopheochromocytoma is a pheochromocytoma-like syndrome characterized by paroxysmal hypertension, abrupt elevation of BP, and the absence of panic at the onset of attack [41]. Many patients with pseudopheochromocytoma suffer from sleep disorders such as poor sleep quality due to activation of the sympathetic nervous system and RAS [42]. Because the quality and quantity of sleep are critical issues for BP elevation in patients with RH, clinicians must consider these issues. Clinical evidence suggests that rapid heart rate that is disproportionate to patients’ BP is also associated with sleep quality [43].

### Secondary hypertension

Secondary hypertension should be evaluated to identify its cause and appropriate treatment in patients with RH (Tables 2 and 3).

**Table 2:** Secondary causes of hypertension

Obstructive sleep apnea
Primary aldosteronism
Renal parenchymal disease
Renal artery stenosis
Pheochromocytoma
Cushing syndrome
Coarctation of the aorta
Hyperthyroidism
Hypothyroidism
Acromegaly

**Table 3:** Laboratory examination of secondary hypertension

Disease	Clinical history	Physical examination	Basic laboratory findings	Screening test	Confirmatory additional test
Renal parenchymal disease	Urinary tract infection or obstruction, analgesic abuse, familial history of polycystic kidney disease	Abdominal mass (polycystic kidney disease)	Proteinuria, hematuria, pyuria, reduced GFR	Renal ultrasound	Further studies for kidney diseases
Renal artery stenosis	Fibromuscular dysplasia, premature hypertension (female), atherosclerotic diseases, sudden onset or worsening of hypertension, resistant hypertension, recurrent pulmonary edema	Abdominal bruit	Rapid worsening of renal function [spontaneous or after ACE inhibitor or ARB treatment]	Kidney size difference > 1.5 cm, Duplex Doppler US, CT	MRI, digital subtraction angiography
Primary aldosteronism	Muscle weakness, premature hypertension, familial history of premature stroke (< 40 years)	Arrhythmia (severe hypokalemia	Hypokalemia (spontaneously or after treatment by diuretics), incidental adrenal mass	Aldosterone-renin ratio (after correction of hypokalemia and excluding effect of ACE inhibitor or ARB)	Suppression test by saline infusion, fludrocortisone, and/or captopril); adrenal CT, adrenal vein sampling
Pheochromocytoma	Paroxysmal hypertension, emergency visit by persistent hypertension with headache, sweat, and/or pallor, familial history	Café-au-lait lesion and neurofibromatosis neurofibroma	Incidental adrenal mass (extraadrenal mass in some cases)	Metanephrine and/or normetanephrine in 24-h urine	Abdominal and/or pelvic CT or MRI; radioisotope scan using meta-iodobenzylguanidine
Cushing syndrome	Rapid weight gain, polyuria, polydipsia, psychiatric problems	Central obesity, moon face, buffalo hump, abdominal striae, hirsutism	Hyperglycemia	Free cortisol in 24-h urine	Dexamethasone suppression test
Hypothyroidism	Dry skin; cold intolerance; constipation; hoarseness; weight gain	Delayed ankle reflex; periorbital puffiness; coarse skin; cold skin; slow movement; goiter		High TSH; low or normal free T4	
Hyperthyroidism	Warm, moist skin; heat intolerance; nervousness; tremulousness; insomnia; weight loss; diarrhea; proximal muscle weakness	Lid lag; fine tremor of the outstretched hands; warm, moist skin		Low TSH; high or normal free T4 and T3	Radioactive iodine uptake and scan
Hypercalcemia and primary hyperparathyroidism	Hypercalcemia	Nonspecific	Elevated serum calcium	Serum calcium	Serum parathyroid hormone
Acromegaly	Acral features; enlarging shoe, glove, or hat size; headache; visual disturbances; diabetes mellitus	Acral features; large hands and feet; frontal bossing		Serum growth hormone ≥1 ng/mL during oral glucose loa	Elevated age- and sex-matched IGF-1 level; MRI scan of the pituitary

*Abbreviation*: *GFR* Glomerular filtration rate, *ACE* Angiotensin II converting enzyme, *ARB* Angiotensin II receptor blocker, *US* Ultrasound, *CT* Computed Tomography, *MRI* Magnetic Resonance Imaging, *TSH* Thyroid stimulating hormone, *IGF* Insulin growth factor

#### Chronic kidney disease

CKD develops into hypertension via various pathogenesis. The upregulated RAS, increased salt and fluid retention, endothelial dysfunction, and increased sympathetic nervous system activity have been suggested as mechanisms of hypertension in CKD [44]. In a previous cohort study, more than 85% of patients with chronic renal disease had hypertension at baseline [45]. Underlying renal parenchymal diseases, such as glomerulonephritis, can also cause hypertension through a similar pathogenesis [46]. Hypertension is a common manifestation of focal segmental glomerulosclerosis, membranous nephropathy, immunoglobulin A nephropathy and membranoproliferative glomerulonephritis [47]. Therefore, kidney disease should be evaluated and considered a risk factor for RH.

#### Primary aldosteronism

Excess aldosterone secretion in primary aldosteronism leads to salt and water retention and renal potassium wasting, resulting in hypertension [48]. Primary aldosteronism is more common than expected, and its prevalence varies from 8 to 30% [49]. Plasma aldosterone-to-renin ratio is the most commonly used screening test [50]. Although there is the potential for false-positive and false-negative results depending on the situation (medications interfering with the RAS, the cutoff values used, the time of testing, and the body positioning at the time of testing), further investigation is required if the aldosterone-to-renin ratio exceeds 20 [48].

#### Obstructive sleep apnea

Obstructive sleep apnea (OSA) is very common, with prevalence rates of 70 - 90% in patients with RH [51]. The high prevalence rates have been attributed to endothelial reactivity, inflammation, oxidative stress, and increased sympathetic and renin-angiotensin-aldosterone system activities, which ultimately lead to increased vascular tone and hypertension [4]. In addition, excess aldosterone and high dietary sodium intake can increase fluid retention and upper airway edema, accompanied by increased airway resistance, thereby worsening OSA [52, 53]. Shifting of fluid from the lower extremities to the neck during supine sleep has been shown to contribute to upper airway edema [54]. This effect is blunted by use of spironolactone [55]. Although routine surveillance by polysomnography is not indicated for all patients with RH, they should be thoroughly screened if OSA is present [56].

#### Renovascular hypertension

Renovascular hypertension (RVH) is a syndrome in which BP is elevated due to renal artery stenosis, leading to renal ischemia. RVH is one of the most common causes of RH, particularly in the elderly [57], with up to 35% of cases being a secondary cause of hypertension [58]. Most cases are caused by atherosclerosis of the renal arteries; however, RVH syndrome can also result from less common obstructive lesions, such as fibromuscular dysplasia, renal artery infarct or dissection, Takayasu arteritis, radiation fibrosis, and renal artery obstruction by an aortic endovascular stent graft [4]. Renal artery stenosis is diagnosed using duplex ultrasonography, computed tomography angiography, or magnetic resonance angiography.

#### Catecholamine-secreting tumors

Catecholamine-secreting tumors, such as pheochromocytomas and paragangliomas, are rare in hypertensive populations accounting for 0.01–0.2% [4]. The prevalence rate is up to 4% in patients referred for RH [59]. Although the prevalence of catecholamine-secreting tumors is low, the mortality and morbidity are high when untreated [60]. Therefore, the diagnoses of patients referred for RH must be considered [61]. Current Endocrine Society guidelines recommend screening by measuring either plasma free (sensitivity, 96-100%; specificity, 89-98%) or 24-hour urine fractionated (sensitivity, 96-100%; specificity, 89-98%) metanephrines. Imaging techniques, such as computed tomography, magnetic resonance imaging, and metaiodobenzylguanidine scanning, should be performed only after biochemical evidence of pheochromocytoma has been obtained [62].

#### Cushing disease or syndrome

Cushing syndrome is a relatively uncommon cause of RH caused by hypercortisolism due to glucocorticoid excess. Cushing syndrome is a constellation of classic symptoms (mood disorders, menstrual changes, and muscle wasting) and signs (acne, osteoporosis, hirsutism, weight gain, moon facies, and supraclavicular fat). However, since 26.5% of patients with Cushing syndrome with biochemical evidence of hypercortisolism do not have overt symptoms [63], clinicians should consider screening tests in patients with RH, even if they do not have the classic syndrome. Current guidelines recommend screening by measuring the 24-hour urine cortisol level, late-night salivary cortisol level, or low-dose dexamethasone suppression test.

#### Other endocrinopathies

Less common endocrine disorders that contribute to RH include thyroid and parathyroid glands [4]. Thyroid-stimulating hormone (TSH) levels should be assessed in patients with difficult-to-control hypertension. Testing for primary hyperparathyroidism should be considered in patients presenting with hypercalcemia.

### Blood pressure measurements

#### Pseudo-resistant hypertension

Many cases of uncontrolled hypertension are not true RH. Despite the use of ≥3 antihypertensive drugs, BP levels may be misrepresented as uncontrolled hypertension—this is known as pseudo-resistant hypertension [4]. The prevalence of pseudoresistant hypertension among patients with aTRH has been estimated to be as high as 50% [64]. The most common causes of pseudoresistance are poor BP measurement, the white-coat effect (WUCH), medication non-adherence, and under-treatment [4]. Thus, physicians should consider excluding pseudo-resistant hypertension before confirming true RH [4].

#### Improper blood pressure measurement technique

Accurate BP measurements can exclude RH. Common errors during routine office BP measurements include not resting for 5 minutes before BP measurement in a quiet room, not using validated devices, not using the correctly sized cuff, not placing the cuff at the heart level, not obtaining a minimum of two readings 1 min apart, deflating the cuff too fast or too slow, observer bias, and auscultatory gap in the auscultation technique [4, 63]. Bhatt et al. reported that improper BP techniques overestimated the prevalence of uncontrolled RH in approximately 33% of patients [64]. In patients with advanced age and atherosclerotic disease, cuff pressure is elevated compared with true intra-arterial measurements; this so-called pseudo-hypertension has been described as a cause of RH. A positive Osler sign, which is a palpable radial pulse while the BP cuffs are inflated above the systolic BP, can be used as a simple screening tool for pseudohypertension. A definitive diagnosis is made by comparing the intra-arterial pressure with the cuff pressure [4].

#### Ambulatory Blood Pressure Monitoring (ABPM)

Out-of-office BP measurements, including ABPM and HBPM, are recommended to exclude true RH [4]. ABPM has been indicated as an essential tool for RH in the following clinical conditions: (1) confirmation of the diagnosis of RH, (2) detection of WUCH, (3) assessment of non-dipping patterns, (4) estimation of cardiovascular prognosis, and (5) assessment of treatment effectiveness in RH [4]. The Spanish ABPM Registry reported that RH was present in 12% of the treated hypertensive population, but more than one-third had normal ambulatory BP (WUCH) [4, 9]. Since non-dipping patterns are commonly observed in patients with RH and nighttime BP levels and dipping patterns provide prognostic value, information on nocturnal BP is important for the diagnosis and monitoring of RH [4]. Claudia et. al. reported that the mean cumulative ambulatory BPs during follow-up compared with the baseline BPs was the best prognostic marker of adverse cardiovascular outcomes and mortality in patients with RH. Therefore, serial ABPM examinations are widely used in RH management [20].

#### Home Blood Pressure Monitoring (HBPM)

HBPM is the best method for diagnosing WUCH and is more convenient, less disruptive, and more cost-effective than ABPM [4]. Some patients are sensitive to ABPM and may experience increased BP readings due to the test itself. Furthermore, ABPM has been linked to insomnia and decreased compliance in some individuals [4]. By contrast, HBPM provides more accurate readings than BP measured in a medical setting and has similar accuracy to ABPM in diagnosing RH [4]. However, HBPM may have limitations in measuring nighttime BP. With recent technological advancements, it is becoming increasingly possible to accurately measure night-time BP at home, and the role of HBPM in the diagnosis of RH is expected to grow [4]. HBPM is a reliable alternative to ABPM for diagnosing RH.

#### White Coat Uncontrolled Hypertension (WUCH)

WUCH is a term used to describe a condition in which a patient’s BP is elevated when measured in a medical setting (such as a doctor’s office) but is normal when measured outside of that setting (such as at home or during daily activities) despite taking antihypertensive medications [8]. WUCH is relatively common, and its prevalence has been reported to be 30-40% in Western countries [9, 65] and 13.5% in Korea [66]. Since WUCH is a major cause of pseudo-resistant hypertension and the white-coat effect is exaggerated in patients with RH [9, 65], its identification is a key factor in the proper diagnosis and planning of the treatment of RH. In the Spanish ABPM Registry, among 8,295 patients diagnosed with RH based on office BP, only 62.5% were classified as having true RH, and the remaining 37.5% had WUCH [9, 65]. Approximately one-third of patients with suspected RH have WUCH [67, 68]. Cardiovascular risk does not increase in patients with WUCH [69]. If WUCH is not excluded and a patient is diagnosed with RH based only on elevated BP measurements in the medical setting, it may lead to unnecessary treatment of high BP with medication, which can have side effects. An accurate diagnosis of WUCH requires out-of-office BP measurements like HBPM or ABPM [4].

### Evaluation of resistant hypertension

#### Medical history

A detailed and comprehensive medical history is essential for evaluating patients with RH as it can provide valuable information for the selection of appropriate treatment strategies. When RH is suspected, the first step is to confirm proper antihypertensive medication adherence by determining the appropriate dose and frequency of administration. Medication adherence can be assessed by checking how often antihypertensive medications are skipped or using self-reported medication adherence assessment tools. If drug discontinuation is confirmed, it is important to identify the reasons for discontinuation, such as side effects and ineffectiveness [70]. Lifestyle factors that may also contribute to elevated BP and should be assessed. These include physical inactivity, high salt intake, smoking, alcohol consumption, and OSA [4]. Healthcare providers should ask about any history of snoring, daytime sleepiness, or witnessed apneas, as OSA is a common comorbidity in patients with RH [51, 71]. Recent changes in weight, stress levels, or sleep patterns should be noted. It is also vital to consider medications that may elevate BP, such as NSAIDs, contraceptives, corticosteroids, and immunosuppressants like cyclosporine. Table 1 summarizes the key items to be checked during the medical examinations of patients with RH. A thorough evaluation is necessary to identify the underlying causes of secondary hypertension in patients with RH (Table 2) [4]. The likelihood of a secondary cause related to RH is higher among older adults. Physicians should conduct a comprehensive assessment to identify underlying causes or contributing factors, such as sleep apnea, renal parenchymal disease, renal artery stenosis, or primary aldosteronism [72].

### Physical examination

During the physical examination of patients with RH, identifying evidence of target organ damage and secondary hypertension is important [4]. BP measurement must be standard and accurate. Coarctation of the aorta is suspected if the difference in BP between the upper and lower extremities is > 10 mm Hg. Evidence of vascular damage can be verified through fundus examination and stenosis of the carotid, and abdominal arteries can be assessed by auscultation of the bruits. Stenotic lesions of blood vessels can also be estimated by palpating the pulsations of the limb arteries. Cushing syndrome can be suspected if there is a moon face or abdominal obesity. If the limbs are enlarged, the lower jaw is lengthened, and the bridge of the nose shows characteristic findings, acromegaly should be suspected, and further examination should be performed.

### Out-of-office blood pressure monitoring

White coat effect may be suspected when BP measurements taken in the clinic are higher than those taken at home, when there is no evidence of hypertension-mediated organ damage despite sustained high BP, and when hypotensive symptoms occur frequently with drug use. The most effective way to rule out the white-coat effect is to conduct out-of-office BP monitoring using ABPM and HBPM. If the white-coat effect is confirmed, out-of-office BP monitoring is recommended for future hypertension treatment [73]. HBPM is particularly useful for diagnosing and managing RH, as it is easy to measure, can be repeated over a long period, and facilitates medication adjustment and improved adherence [74].

### Laboratory examination and imaging

Laboratory examinations for the evaluation of RH should include basic blood chemistry (serum sodium, potassium, chloride, bicarbonate, glucose, blood urea nitrogen, and creatinine with glomerular filtration rate(GFR and urinalysis. Plasma aldosterone and plasma renin activity should be evaluated to screen for primary aldosteronism. Interpretation of the aldosterone-to-renin ratio is difficult in patients who take certain antihypertensive medications, such as mineralocorticoid receptor antagonists, which increase aldosterone levels, or direct renin inhibitors and beta-blockers, which lower renin levels [75, 76]. The elevated aldosterone-to-renin ratio(ARR) show low specificity because low-renin states are common (eg, volume expansion, dietary salt excess or sensitivity). A high ratio (> 20) when the serum aldosterone is >16 ng/dL and PRA is <0.6 ng/mL per hour is suggestive of primary aldosteronism, particularly in a patient taking an ACE inhibitor or ARB, but further assessments are required to confirm this diagnosis. Measurement of plasma metanephrines or 24-hour urinary metanephrines is an effective screening test for patients with suspected paraganglioma or pheochromocytoma [77]. Thyroid function tests, such as free T4, T3, and TSH are useful for screening for hyperthyroidism and hypothyroidism [2].

Imaging tests for screening renovascular hypertension are performed in young patients with a likelihood of fibromuscular dysplasia and in older patients with a history of smoking or vascular disease who have an increased risk for atherosclerosis [2]. Screening tools for renovascular hypertension include a captopril renal scan, Doppler ultrasound, computed tomography, or magnetic resonance angiography. Patients with hypokalemia without an apparent etiology or an incidentally diagnosed adrenal mass should be evaluated for hyperaldosteronism. Paroxysmal and/or refractory hypertension with hyperadrenergic symptoms is indicative of a strong possibility of pheochromocytoma. Therefore, after the measurement of aldosterone, catecholamine, and cortisol levels in the plasma and/or 24-h urine, abdominal imaging of the adrenal glands by high-resolution computerized tomography, magnetic resonance imaging, or radioisotope imaging (I-131 metaiodobenzylguanidine) is indicated if there is biochemical evidence of hormonally active tumors [5].

Sleep apnea syndrome has been suggested to be a prevalent cause of secondary hypertension among obese patients or those with resistant hypertension. Although there is still no evidence of its benefits, screening for sleep apnea using a self-questionnaire or diagnosis using sleep polysomnography may be useful for the evaluation of RH [10].

### Management of resistant hypertension

Confirming aTRH and screening diagnostic issues like non-adherence, accuracy of BP measurement, and the exclusion of temporary factors such as lifestyle factors, related drugs or herbal medication is critical when deciding upon referral to a specialist.

### Adherence intervention

Non-adherence to antihypertensive medication is a key contributor to uncontrolled BP; previous studies have indicated that over 30% of adults taking antihypertensive medication have low adherence in the year following treatment initiation [78]. Meta-analysis of the prevalence of non-adherence among adults with aTRH in 42 studies with 71,353 participants showed an average prevalence of 37%, with wide variation across the studies ranging from 3–86% [79].

Antihypertensive medications can be assessed using both indirect and direct methods [80]. Indirect methods include questionnaires, diaries, pill counts, and prescription refill rates. Direct methods include directly observing therapy (i.e., watching people take each dose of medication) and measuring metabolite levels or biological markers [81]. Generally, the prevalence of non-adherence is substantially lower when adherence is ascertained using indirect methods (20%) compared with direct methods (46%) [79]. Practically, a single-pill combination of antihypertensive medications and statins is helpful for serially checking adherence by evaluating cholesterol levels. The direct method provides an accurate assessment of medication taken recently but does not capture long-term adherence and is not available in every center.

Several factors, including socioeconomic status, demographics, and environmental factors, are associated with suboptimal adherence. More importantly, the rapport between the patient and clinician, the communication style of the clinician, and the degree of shared decisions have all been shown to affect adherence [80]. Access to and cost of medications are clearly important for adherence, although the nearly full coverage of the national health insurance system in Korea has an advantage over other countries. Complex medication regimens, including polypharmacy and multiple daily doses, reduce adherence. Finally, adults with hypertension often have multiple chronic conditions, including depression and cognitive dysfunction, which can adversely affect their adherence to medications and healthy lifestyles.

Drug adherence is an important but often neglected factor in the evaluation of patients with RH. According to the American Heart Association, confirmation of RH requires the assurance of antihypertensive medication adherence [4]. This is due to the high prevalence of medication non-adherence in RH, which has been reported to be as high as 50%. In a study by Lawson et al., 300 patients with uncontrolled hypertension underwent a urine antihypertensive drug assay using liquid chromatography-tandem mass spectrometry (LC-MS/MS). The study showed that 55% of the patients were nonadherent to the prescribed medications [82]. In a study of 76 patients with uncontrolled RH, 40 (53%) were found to be non-adherent [83]. The prevalence of non-adherence is similarly high in patients with refractory hypertension. In a study by Siddiqui et al., 54 patients with apparent refractory hypertension underwent a urine antihypertensive drug metabolite assay using high-performance liquid chromatography-tandem mass spectrometry. The results showed that of the 40 fully evaluated patients, 16(40.0%) were fully adherent, 18(45.0%) were partially adherent, and six (15.0%) were non-adherent [84]. Despite the importance of medication adherence, it is difficult to assess in clinical practice. The current limitation in assessing medication adherence is the lack of a standardized, clinically available method or test to accurately assess medication adherence. Pill counting, the most widely used method in clinical trials, is associated with an overestimation of drug adherence. Electronic monitoring and drug metabolite assays, either in the blood or urine, are investigational methods that are not widely available [85, 86]. Efforts to improve medication adherence are important. First, patient education to improve self-awareness of medical conditions and the need for proper ingestion of antihypertensive medications is important. Physician efforts to lower pill counts are also important, as studies have shown a significant inverse association between antihypertensive pill counts and medication adherence [87]. Several trials designed to reduce non-adherence to antihypertensive medications have been conducted, however, their effects vary. Although a trial of simple reminder pill bottles did not show an improvement in adherence, using electronic pill devices that trigger text message reminders in conjunction with missed doses shows promise in improving adherence [88]. Morawski et al. randomized patients with poorly controlled hypertension to usual care or a smartphone app that provided reminder alerts, adherence reports, and optional peer support and found that patients receiving the intervention had improved self-reported adherence, but the intervention did not affect BP control [89]. Lastly, simplifying treatment regimens by consolidating doses using fixed-dose combinations or extended-release formulations is reportedly effective in improving adherence [90]. Switching a patient prescriptions to a once-daily regimens using fixed-dose combinations to reduce the total pill count may help improve medication adherence in patients with RH.

### Lifestyle factor intervention

Healthy lifestyles, including low sodium intake, regular exercise, ideal body weight, moderate alcohol intake, and non-smoking, are important for promoting public health and preventing the development of hypertension and cardiovascular events [22, 91]. All physicians managing RH should review patients’ lifestyles and recommend maintaining the following healthy lifestyles.

#### Diet

Dietary sodium intake is closely associated with BP [29]. Experimental and epidemiological studies have shown that increased salt consumption increases BP, resulting in incident hypertension and cardiovascular complications [92, 93]. Reducing sodium intake lowers BP [29]. The American Heart Association defines sodium sensitivity as a characteristic that affects BP according to changes in salt intake [94]. BP in sodium-sensitive individuals responds more sensitively to sodium intake than that in sodium-resistant individuals. Sodium sensitivity is higher in hypertensive populations than in general populations without hypertension, and higher in resistant hypertensive patients than in simple hypertensive individuals [94, 95]. Older adults and people with a higher level of BP or comorbidities, such as CKD and diabetes, tend to be more sensitive to sodium intake [22]. A randomized crossover trial reported that low-salt diet decreased systolic and diastolic BP by 22.7 and 9.1 mmHg, respectively, in patients with RH [32]. To control BP and prevent cardiovascular complications, salt and sodium restrictions are recommended for patients with RH.

Dietary patterns significantly affect the incidence of chronic diseases, including hypertension and diabetes. The Dietary Approaches to Stop Hypertension (DASH) trials emphasized that hypertensive people consume more fruits, vegetables, and low-fat dairy foods and less red meat, saturated or total fat, and sugar-containing beverages to reduce their BPs [96]. The DASH diet reduced systolic and diastolic BP by 5.5 and 3.0 mmHg, respectively [96].

For patients with RH, adopting a DASH diet and reducing salt (6 g/day) and sodium (2.4 gram/day) intake are recommended to control BP [8].

#### Physical activity

Low physical activity and fitness levels were independent risk factors for hypertension. Physical activity is inversely associated with the development of hypertension [97]. Physical activity reduces BP in both hypertensive and normotensive populations, independent of weight loss [98]. In a meta-analysis, BP lowered by approximately 8.3 mmHg and 5.2 mmHg in systolic and diastolic BP over several endurance (aerobic) exercise programs in hypertensive population [99]. Resistance (muscle strengthening) exercises also reduce BP in patients with an elevated BP [99]. Regular physical activity is inversely associated with cardiovascular and/or all-cause mortality in patients with elevated BP [100]. Thus, physical activity is beneficial for reducing mortality in hypertensive populations in addition to BP reduction. Clinical practice guidelines for hypertension from Europe, America, and Asia commonly recommend regular physical activity [8, 22, 91]. However, there are few studies for patients with RH to follow current exercise recommendations. In patients with RH, regular aerobic exercise based on a treadmill and heated water pools reduces systolic and diastolic BP and improves physical performance [34, 101]. Another study demonstrated that a 12-week aerobic exercise training improved central BP, BP variability, and cardiovascular biomarkers such as interferon-gamma and angiotensin II in resistant hypertensive patients [102]. Despite the lack of evidence showing an association between exercise and BP in patients with RH, the current recommendations developed by experts are appropriate. In general, it is recommended that patients with RH engage in at least 150 min of moderate-intensity or 75 min of vigorous-intensity aerobic exercise per week [8, 22, 91]. A gradual increase in aerobic exercise to 300 min of moderate-intensity or 150 min of vigorous-intensity aerobic exercise is advised [103]. Muscle-strengthening exercises involving all major muscle groups for 2 or more days per week and balance training may also be advised [104].

#### Weight control

Body mass index is positively correlated with increased BP [26]. Patients with increased adiposity usually have insulin resistance, high sympathetic nervous system activity, an activated RAS, and increased salt sensitivity [28]. Elevated insulin levels stimulate sympathetic tone and the renin-angiotensin-aldosterone system. In obese individuals, sodium is reabsorbed by the kidneys via renovascular mechanisms [105]. Of these complicated pathophysiological mechanisms, obese patients are likely to develop RH [26, 28]. A long-term excessive energy intake of as little as 50-100 kcal per day, even in small increments of sugars, refined grains, processed foods, and alcoholic beverages when consumed for a long time, causes weight gain [106, 107]. These common risk factors for obesity and hypertension can also cause BP elevation and treatment-RH. Thus, weight reduction through a healthier lifestyle is helpful for improving BP control. Systematic reviews consistently demonstrated that 1 kg of weight reduction decreased systolic BP by approximately 1 mmHg during a follow-up of 2-3 years even although this inverse association was attenuated in longer-term follow-up [108–110]. Guidelines strongly recommend that overweight or obese adults with elevated BP or hypertension should lose body weight to the ideal body weight for BP reduction [8, 22, 91].

#### Alcohol consumption

Although modest alcohol intake increases high-density lipoprotein cholesterol levels and is inversely associated with a lower risk of coronary heart disease than abstinence [111, 112], excess alcohol intake has harmful effects on BP and cardiovascular events. Excessive alcohol consumption increases BP and accounts for approximately 10% of the population with hypertension. Patients with hypertension who consume heavy alcohol are more likely to be resistant to antihypertensive management, resulting in RH. According to a meta-analysis of randomized controlled trials, reduced alcohol consumption significantly decreased the mean systolic and diastolic BP in a dose-dependent manner [113, 114]. Adults with elevated BP or hypertension who currently consume alcohol should be advised to drink 2 and 1 or less standard drinks per day in men and women, respectively [8, 113–115]. One standard drink contained approximately 14 g of pure ethanol [116].

#### Tobacco smoking

Tobacco smoking and secondhand smoke are modifiable risk factors for hypertension and cardiovascular events, in addition to various malignant neoplasms and chronic obstructive pulmonary disease (COPD). Tobacco smoking causes an immediate but prolonged pressor effect, which may increase daytime ambulatory BP and lead to inaccurate results [117, 118]. However, several studies have reported that BP is lower in current smokers than in non-smokers or former smokers, and smoking cessation is significantly related to hypertension risk [119]. Transient elevation in BP after smoking cessation may have been caused by weight gain. The health benefits of smoking cessation outweigh the harmful effects, such as transient BP elevation through weight gain [120]. Tobacco smoking threatens public health, accounting for nearly 6.3 million deaths and 6.3% of global disability-adjusted life years (DALYs) [121]. To ameliorate the public health burden of tobacco smoking, all patients with hypertension who smoke tobacco should be advised to stop smoking.

### Pharmacologic management of resistant hypertension

For pharmacological management of RH, a combination of optimal or maximally tolerated doses of long-acting RAS inhibitors, calcium channel blockers, and thiazide-type or thiazide-like diuretics should be prescribed [4, 22, 91]. If a patient has uncontrolled BP despite maximum tolerated triple combination consider switching RAS inhibitors and calcium channel blockers to the most potent, longest acting drugs in each class and to switch thiazide type diuretics to thiazide like diuretics such as chlorthalidone and/or indapamide [4]. Thiazide-like diuretics have more potent BP-lowering effects than hydrochlorothiazides [122]. Recommendations have suggested that in patients with CKD with an estimated GFR < 45 ml/min/m^2^ thiazide diuretics may not be effective and loop diuretics should be used instead. However, increasing evidence suggests that thiazide like diuretics can effectively reduce BP in patients with low eGFR [123]. The CLICK trial demonstrated that chlorthalidone is effective in lowering BP in patients with advanced CKD and uncontrolled hypertension [124]. This is important, considering the high prevalence of RH in patients with CKD [125]. Because it is unavoidable that patients with RH are prescribed a higher number of antihypertensive medications, RH may be associated with poor drug adherence [10]. Therefore, medications should be minimized by considering a fixed-dose combination at the beginning and, if not, by considering switching the medications to a fixed-dose combination when stabilized. In patients with CKD stage 4 or stage 5(eGFR < 30 ml/min/1.73 m^2^) and either resistant edema or uncontrolled BP, loop diuretics should be considered and can be used with existing thiazide diuretics [126]. Furosemide, which has a short half-life, is administered twice daily, whereas torsemide may be administered once daily.

When BP is not controlled by the maximally tolerated triple combination of RAS inhibitors, CCBs, and diuretics, spironolactone is considered the drug of choice. In the PATHWAY 2 trial, spironolactone demonstrated significantly better efficacy than doxazosin and bisoprolol in reducing the primary endpoint of the home systolic BP in patients with RH [127]. In cases where spironolactone is not tolerated, potassium-sparing diuretics, such as amiloride, may be considered [128]. However, no randomized clinical trials have compared the efficacy of spironolactone and amiloride in patients with RH. The major limitation of potassium-sparing diuretics is the relatively high incidence of hyperkalemia. This is even more problematic in patients with resistant hypertension owing to the high prevalence of CKD. In the AMBER trial, the use of spironolactone in patients with CKD and RH treatment, with or without the concomitant use of a potassium binder, was 64.2% and 35.4%, respectively [129, 130]. This study showed that using a potassium binder (patiromer) in advanced CKD patient with RH can allow for more patients to be maintained on spironolactone. Non-steroidal aldosterone antagonists, which are associated with a lower incidence of hyperkalemia than spironolactone, appear promising [130]. If BP is not controlled with spironolactone or if spironolactone cannot be used, the sequential addition of beta-blockers, alpha-blockers, and vasodilators(hydralazine, minoxidil) should be considered [4]. This treatment regimen should be sufficient to control BP in most patients with RH. However, less than 1% of patients with hypertension (10% of patients with RH) are refractory to medical treatment, including thiazide-like diuretics and spironolactone and are considered to have refractory hypertension.

Managing RH in older adults requires a comprehensive approach. Older adults are more likely to take medications that increase their BP. Therefore, efforts should be made to identify medications, such as NSAIDs, steroids, or herbal medications, that may contribute to uncontrolled hypertension. Other treatment strategies may involve the optimization of medication regimens by adjusting dosages, adding medications from different drug classes, or using fixed-dose combinations. Lifestyle modifications, including weight loss, regular exercise, and a low-sodium diet are also important in older patients. However, strict lifestyle modifications can lead to dehydration, sarcopenia, or fall-related injuries; thus, individualized recommendations should be provided to older patients with hypertension [131].

### Non-pharmacologic (Device Based) management of resistant hypertension

The pivotal role of the sympathetic nervous system in the development and progression of hypertension is well-established [132]. Before the availability of antihypertensive medications, surgical sympathectomy was used [133]. It has since been largely phased out owing to undesirable side effects such as orthostatic hypotension and the growing availability of antihypertensive medications. Despite its discontinuation, significant strides made in BP control following surgical sympathectomy have served as a foundation for the development of device-based treatments for RH.

#### Renal denervation

Renal denervation (RDN) has emerged as a promising novel intervention for RH management [4]. The procedure targets the renal sympathetic nerves, which play a pivotal role in the regulation of BP, by delivering radiofrequency, ultrasound energy, or ethanol to interrupt the efferent and afferent nerves in the renal arteries.

The early promise shown in a proof-of-concept study [134] (SYMPLICITY HTN-1) and a subsequent randomized open-label study [135] (SYMPLICITY HTN-2) was tempered by the results of the SYMPLICITY HTN-3 trial [136], which failed to meet its primary efficacy endpoint in the RH population. However, the methodological concerns arising from SYMPLICITY HTN-3, including alterations in patient behavior in adherence to their multidrug pharmacological regimens and inadequate denervation resulting from technical failure of the catheter and/or the interventional proceduralist have led to several new catheters and new trial designs to evaluate the efficacy of more extensive renal denervation, and subsequent studies have presented more favorable results.

Several recent sham-controlled clinical trials [137–139] (SPYRAL HTN-OFF MED, RADIANCE-HTN SOLO, RADIANCE II trial) in patients with mild to moderate HTN, although not studies in patients with RH, have shown significant reductions in BP in RDN group. Based on these results, RDN has emerged as an evidence-based option for treating hypertension by complementing lifestyle modifications and antihypertensive medications [140].

More recent randomized controlled trials [141–143] (DENERHTN, SPYRAL HTN-ON MED, and RADIANCE-HTN TRIO trials) have demonstrated significant BP-lowering effects of RDN in uncontrolled hypertension, renewing optimism about the therapeutic potential of this approach. In the SPYRAL HTN-ON MED trial, 80 hypertensive patients uncontrolled with one to three antihypertensive medications were randomized to the Symplicity Spyral multielectrode catheter ablation or sham control group. The mean adjusted difference for the primary endpoint of 24 h systolic BP at 6 month was -7.0 mmHg(95% CI: -12.0 to -2.1, *P*=0.0059) [142]. In the long term follow up study of the SPYRAL HTN-ON MED trial, the adjusted difference for the 24 hour systolic BP at 36 months was -10.0 mmHg(95% CI: -16.6 to -3.3, *P*= 0.0039) [144]. The results demonstrate that the efficacy of renal denervation may be maintained in the long-term. In the RADIANCE-HTN TRIO trial, 136 patients with RH were randomized to receive renal denervation with an endovascular ultrasound device or a sham procedure, with the primary endpoint being the change in daytime ambulatory systolic BP at 2 months. The results showed a significant difference in the daytime systolic BP [median difference 4.5 mmHg(95% CI, -8.5 to -0.3, *P*=0.022)] [143], demonstrating the efficacy of renal denervation in treatment RH. However, despite these promising results, further long-term studies are needed to fully elucidate the role of RDN in the management of RH and identify the patient subgroups most likely to benefit. Considering the modest reduction in BP, more data are needed regarding the cost-benefit of renal denervation in patients with RH. A comparative study of aldosterone antagonists, their efficacy in refractory hypertension, and their efficacy in patients with CKD should be conducted. In the 2023 European Society of Hypertension guidelines for the management of arterial hypertension, renal denervation was given a class II recommendation for the treatment of RH with an eGFR > 40 ml/min/1.73 m^2^ [145].

#### Baroreflex activation therapy

The carotid baroreceptor reflex regulates the BP. Carotid baroreceptor activation therapy (BAT) involves the implantation of a device near the carotid sinus that modulates autonomic outflow. This modulation leads to a reduction in sympathetic activity and an increase in parasympathetic activity, resulting in decreased BP [146]. Endovascular baroreflex amplification is a novel technique used to lower BP in RH. The BAT is a crucial aspect of baroreflex amplification that specifically targets the carotid sinuses. BAT can be categorized into electrical and mechanical types.

Currently, two generations of electrical BAT have been developed and are supported by evidence from clinical trials. The first-generation Rheos System, which was evaluated in the Rheos Pivotal trial [147], did not receive FDA approval because of safety concerns. In contrast, the second-generation Barostim Neo System, assessed in the Barostim Neo Trial [148] received FDA approval for the treatment of heart failure. A recent cohort study with the Barostim Neo device demonstrated its efficacy over a period of 2 years, with 25 of 50 patients with RH achieving controlled office systolic BP levels below 140 mmHg [149]. Further randomized controlled trials are required to validate the effects of BAT on RH. However, this novel device has some unmet needs, including invasiveness during the procedure, concerns about battery replacement, and potential side effects such as syncope, arrhythmias, and unadjusted BP levels.

The less invasive Endovascular Baroreflex Amplification (EVBA) Mobius HD device implanted within the carotid sinus mechanically modulates the baroreceptors by enhancing wall stretch. This modulation activates the carotid baroreceptor and reduces BP levels [150]. The first clinical study was conducted in 2013 and showed a notable reduction in office BP at six months, with systolic and diastolic BP decreasing by 24 mmHg and 12 mmHg, respectively [151]. However, some patients experience hypotension, worsening hypertension, and infections. After a 3-year follow-up period, a significant reduction of 30 mmHg in SBP was observed, demonstrating the efficacy of EVBA in reducing BP with an allowable safety profile [152]. The CALM-2 trial is an ongoing, randomized, double-blind, sham-controlled trial evaluating EVBA using a MobiusHD device. This study enrolled 300 participants and assessed the change in mean 24-hour ambulatory systolic BP at the six-month as the primary endpoint, providing valuable insights into the effectiveness of the therapy [153]. Additional randomized, sham-controlled trials are required to confirm the long-term efficacy and safety of EVBA devices. Close collaboration between interventionists and clinicians plays a pivotal role in determining the success of device therapy in patients with RH.

#### Continuous Positive Airway Pressure (CPAP)

OSA is associated with an increase in nocturnal BP, a non-dipping pattern in diurnal BP variability, and an increase in morning surge, which may contribute to the difficulty of treating hypertension and increase the risk of cardiovascular events [154, 155]. OSA is very common in patients with RH, with the reported prevalence of up to 70 to 90% [4]. This may be due to an increase in fluid retention and upper airway edema in RH, which increases upper airway resistance and predisposes these patients to severe OSA [4, 54, 156]. Overall, the reported effect of continuous positive airway pressure (CPAP) on BP reduction in patients with RH is modest. In a study of 194 patients with RH who were randomized to CPAP or no therapy, CPAP was associated with a significantly greater decrease in the 24-hour mean BP [3.1 mmHg (95% CI, 0.6 to 5.6), *P* = 0.02] and 24-hour DBP [3.2 mmHg (95% CI, 1.0 to 5.4 mmHg), *P* = 0.005] at 12th week of treatment. However, this and other recent clinical trials using CPAP enrolled asymptomatic patients with OSA for obvious ethical reasons, while excluding subjects with symptomatic OSA [157]. This may underestimate the efficacy of CPAP as symptomatic OSA patients with RH may have a more robust response to effective CPAP therapy. In a randomized study of 60 symptomatic patients with moderate to severe OSA who were randomized to effective CPAP versus subtherapeutic CPAP for 9 weeks, the mean arterial BP decreased by 9.9±11.4 mmHg in patients undergoing effective CPAP therapy [158]. Therefore, a careful patient history that assesses the possible symptoms of OSA, such as daytime somnolence and morning headache, should be ascertained in patients with RH. In patients with typical symptoms, testing for OSA is highly recommended, because CPAP may be effective in improving BP control. Moreover, a prospective observational study on treatment-RH with OSA revealed that BP reduction by CPAP was stronger in patients with nocturnal hypertension evaluated by ABPM compared to that in patients with nocturnal normotension [159]. Therefore, the assessment of the presence of nocturnal hypertension using ABPM is also recommended, especially in patients with treatment-RH complicated by OSA.

### Refractory hypertension

Refractory hypertension is defined as hypertension that cannot be controlled below the target BP with the use of 5 or more antihypertensive agents. A stricter definition is the failure to maintain BP below the target BP with the use of five or more antihypertensive agents, including thiazide-like diuretics(chlorthalidone or indapamide) and spironolactone [160]. In an analysis of 14,809 participants receiving antihypertensive agents from the Reasons for Geographic and Racial Differences in Stroke(REGARDS) Study, 2144(14.5%) had RH and 78(0.5%) had refractory hypertension [6]. Black race, male sex, higher body mass index(BMI), living in the stroke area of the United States(Southern States), lower heart rate, reduced estimated GFR, albuminuria, diabetes mellitus, history of stroke, and history of coronary heart disease were significantly associated with refractory hypertension [6]. In a prospective cohort study of 1,726 patients with RH, the reported prevalence of refractory hypertension among subjects with RH was 8.6% [161]. In the same cohort, patients with refractory hypertension were younger, more obese, and had a higher prevalence of smoking and CKD. Surprisingly, the prevalence of DM was also lower. Patients with refractory hypertension had a significantly higher heart rate than those without refractory hypertension, despite the higher use of beta-blockers [161]. Previous studies have also demonstrated that patients with refractory hypertension are younger, with evidence of higher sympathetic nervous system activation, and no significant difference from non-refractory hypertension in terms of volume excess parameters [160, 162]. This may explain the lack of response to diuretics and aldosterone antagonists.

Refractory hypertension is associated with increased risk of adverse cardiovascular events overall and have increased risk compared to non-refractory RH. In an analysis of a prospective cohort of 1576 patients with RH, refractory hypertension was associated with significant increase in the risk of total cardiovascular events(HR:1.44[1.01-2.07]), major adverse cardiovascular events(HR:1.68[1.16-2.45]), cardiovascular mortality(HR:1.85[1.18-2.90]) and stroke(HR:2.14[1.17-3.93]) [161] compared with subjects with non-refractory RH [161]. In a recent Korean study, 150(0.9%) of 16,284 patients who underwent ABPM had refractory hypertension. Similar to previous reports, patients with refractory hypertension had a higher prevalence of obesity, DM, CKD, heart failure, myocardial infarction, and stroke. For cardiovascular mortality, the adjusted HR of refractory hypertension was 5.22 compared to non-RH [16]. As the treatment options for these subsets of patients are currently limited, the development of treatment options for refractory hypertension is an unmet need in the field of hypertension that warrants further research [163].

## Conclusion

The prevalence of the aTRH is approximately 10-15%. Among these patients, specific aspects, such as pseudo-resistance, WUCH, secondary hypertension, non-adherence, and inappropriate treatment regimens, must be ruled out to properly diagnose true RH. Comprehensive treatment approaches, including lifestyle interventions, pharmacological approaches, and systematic approaches to improve adherence, are needed to control RH. Device-based therapies aimed at sympathetic nervous system activity are currently used in patients with RH. Among device-based therapies, RDNs have been shown to be effective in some patients with RH; however, more research is needed to apply them to a larger number of patients with RH. Further studies are required to determine the long-term efficacy and safety of these devices. Refractory hypertension, defined as the failure to control BP below the target BP with the use of five or more antihypertensive agents, including thiazide-like diuretics (chlorthalidone or indapamide) and spironolactone, is associated with a poor prognosis. Further research is warranted to develop better treatment options for refractory hypertension.

## Data Availability

“Not applicable”

## Abbreviations

BP: Blood pressure
RH: Resistant hypertension
RAS: Renin-angiotensin system
ABPM: Ambulatory blood pressure monitoring
HBPM: Home BP monitoring
CKD: Chronic kidney disease
DM: Diabetes mellitus,
ALLHAT: The Antihypertensive and Lipid-Lowering Treatment to Prevent Heart Attack Trial
aTRH: Apparent treatment resistant hypertension
DASH: Dietary Approaches to Stop Hypertension
NSAIDs: Nonsteroidal anti-inflammatory drugs
OSA: Obstructive sleep apnea
RVH: Renovascular hypertension
WUCH: White-coat uncontrolled hypertension
COPD: Chronic obstructive pulmonary disease
DALYs: Disability-adjusted life years,
EVABA: Endovascular Baroreflex Amplification
CPAP: Continuous positive airway pressure
REGARDS: Reasons for Geographic and Racial Differences in Stroke
ACE: Angiotensin converting enzyme
ARB: Angiotensin II receptor blocker
TSH: Thyroid stimulating hormone
CCBs: Calcium channel blockers
